# A novel cross-species vaccine design and nasal immunization strategy based on recombinant *Lactiplantibacillus plantarum* expressing PEDV tS1

**DOI:** 10.1128/aem.00386-26

**Published:** 2026-05-11

**Authors:** Jiayi Hao, Xieshen Zhou, Guoqing Zhang, Lingcong Deng, Letian Li, Maopeng Wang

**Affiliations:** 1 College of Veterinary Medicine, Northwest A&F University, Yangling, China; 2 Wenzhou Key Laboratory for Virology and Immunology, Institute of Virology, Wenzhou University 26495, Wenzhou, China; 3 State Key Laboratory of Pathogen and Biosecurity, Key Laboratory of Jilin Province for Zoonosis Prevention and Control, Changchun Veterinary Research Institute, Chinese Academy of Agricultural Sciences 595703 , Changchun, China; 4 College of Animal Sciences, Key Laboratory for Zoonoses Research, Ministry of Education, Jilin University 12510, Changchun, China; University of Nebraska-Lincoln, Lincoln, Nebraska, USA

**Keywords:** *Lactiplantibacillus plantarum*, PEDV, intranasal immunization, mucosal vaccines, cross-species vaccination

## Abstract

Porcine epidemic diarrhea (PED) is a highly lethal and airborne disease in neonatal piglets that has caused significant economic losses in the pig industry over the last three decades. Despite the widespread implementation of commercial vaccines, the disease continues to exhibit recurrent outbreaks, primarily owing to suboptimal immunization efficacy. In this study, we engineered recombinant *Lactiplantibacillus plantarum* LP12:PEDV tS1, which expresses the truncated S1 domain of the porcine epidemic diarrhea virus (PEDV) spike protein through a surface anchoring mechanism. The expression was confirmed using western blotting, flow cytometry, and indirect immunofluorescence assays, with a maximum protein yield of 1.7657 μg per 1×10^9^ CFU. Furthermore, we immunized mice and pigs nasally with LP12:PEDV tS1 separately. Immunization elicited robust mucosal immune responses, as evidenced by a significant increase in specific secretory IgA (sIgA) with neutralizing activity in alveolar lavage fluid and fecal samples. Our results also indicated that LP12:PEDV tS1 activated dendritic cells in the mesenteric lymph nodes of mice, marked by notable upregulation of CD80 and CD86. Successive immunization with LP12:PEDV tS1 protected piglets from PEDV infection and elicited comparable mucosal immune responses across species, demonstrating its strong potential as a PEDV vaccine candidate. Moreover, an intranasal immunization strategy based on recombinant *Lactiplantibacillus plantarum* demonstrated suitability for cross-species vaccination between mice and piglets, potentially streamlining the preclinical evaluation process.

**IMPORTANCE:**

Porcine epidemic diarrhea (PED) is a major cause of piglet mortality. Current control relies on vaccinating pregnant sows with inactivated or attenuated viruses. However, not all sows develop sufficient antibodies to protect piglets. Once piglets are infected, no effective emergency vaccines are available for them, as existing options pose biosafety risks. To address this, we developed a subunit mucosal vaccine using recombinant *Lactiplantibacillus plantarum*. This vaccine is cost-effective, enables cross-species immunization, and remarkably provides immediate immunity to prevent viral infection in neonatal piglets.

## INTRODUCTION

The coronavirus family cause various respiratory and gastrointestinal diseases in many diverse species (1–3). Porcine epidemic diarrhea (PED) is an acute and highly contagious disease caused by a member of the coronavirus family called porcine epidemic diarrhea virus (PEDV). The main clinical symptoms are watery diarrhea, vomiting, and dehydration (4). Since 2010, the emergence of GII strains of PEDV has led to outbreaks of severe viral diarrhea in China, South Korea, and Japan, with the mortality rate of 100% in newborn piglets and substantial economic losses to the global swine industry (5, 6). The PEDV genome spans approximately 28 kb and encodes 16 non-structural proteins (NSPs), four structural proteins, and one accessory protein (ORF3)(7). The spike (S) protein, a surface glycoprotein of the virion, is crucial for mediating host cell receptor binding and viral entry, and it is the primary antigen that elicits neutralizing antibodies production against PEDV in the host (8, 9). Correspondingly, as a key antigen for coronavirus vaccine development, the spike protein, especially the S1 subunit, which recognizes receptors and determines virus-host interaction (10), is expected to be an ideal target for the design of anti-PEDV vaccines.

Current PEDV vaccines, including attenuated and inactivated types, are widely used but have limitations (11). Highly efficient airborne transmission and inadequate vaccination prevent vaccinated sows and gilts from eliciting protective lactogenic immunity in their progeny (12, 13). Traditional methods, such as intramuscular injection, trigger systemic immunity but lack mucosal immunity, which is crucial for protection. This discrepancy underscores the importance of considering both systemic and mucosal immune responses in vaccine design (14). Additionally, current vaccines, either attenuated or inactivated, may pose potential biosafety risks (15). Recent advances have focused on next-generation platforms to address these flaws, with mRNA vaccines emerging as a promising candidate. Encapsulated by lipid nanoparticles (LNPs), PEDV mRNA vaccines targeting the spike (S) protein induce robust neutralizing antibodies and cellular immunity, yet face bottlenecks like harsh storage conditions and inadequate mucosal immune activation (16). Furthermore, certain nucleic acid-based and viral vector vaccines heavily rely on species-specific codon optimization, complicating cross-species evaluation and hindering the development and application of these vaccines (17–19). These technical hurdles underscore the need for innovative approaches.

PEDV primarily infects the intestinal mucosa of piglets and replicates in epithelial cells of the small intestine and colon (8). Induction of antigen-specific secretory IgA (sIgA) through mucosal immunity is associated with enhanced protective efficacy against PEDV infection (20–22). Recent studies have elucidated the mechanisms of airborne transmission of PEDV and its capacity to invade the intestinal mucosa via the nasal cavity (23, 24). Following inhalation, PEDV is internalized by dendritic cells residing in the nasal submucosa and then undergoes systemic circulation to reach the intestinal mucosa (24). These findings suggest that immune cells in the nasal mucosa may serve as key targets for vaccine development. It highlights the potential relevance of intranasal immunization for other enteric pathogens. These findings collectively suggest that intranasal immunization represents a promising alternative vaccination strategy for PEDV prevention (25–27).

Gram-positive lactic acid bacteria, such as the *Lactiplantibacillus* genus, have a generally recognized as safe (GRAS) status. Beyond their well-established role in food fermentation and preservation, these microorganisms have garnered significant attention as potential delivery vehicles for heterologous protective antigens owing to their intrinsic immunomodulatory properties and mucosal adherence capabilities (28). This characteristic makes them particularly suitable for the development of mucosal vaccine platforms. In this study, we provided a novel strategy based on the recombinant *Lactiplantibacillus plantarum* (*L. plantarum*) expression system for the development of a PEDV mucosal vaccine. The truncated PEDV S1 was expressed on the surface of *L. plantarum* LP12. The resulting recombinant *L. plantarum* (LP12:PEDV tS1) was administered to evaluate its immunogenicity in mice and piglets. The results showed that it triggered both species’ immune responses, producing pathogen-specific sIgA, a marker of mucosal immunity that is crucial for combating PEDV. Therefore, the findings underscore the potential of *Lactiplantibacillus*-based expression systems as a viable platform for the development of mucosal vaccines, particularly for enteric pathogens, where mucosal immunity plays a pivotal role in protection.

## MATERIALS AND METHODS

### Bacterial strains, plasmid, and animals

*Lactococcus lactis* strain NZ3900, *L. plantarum* LP12, and plasmid pSIP411 were obtained or cultured as previously reported (29). Six-week-old female BALB/c mice (SPF biotechnology, China) were reared in a standard environment without pathogens (light/dark 12 h, temperature 22–25°C, relative humidity 45%–50%) to provide standard food and water. Hybrid piglets (Large White breed) were obtained from a local commercial swine farm, artificially reared in a conventional animal facility, and housed in stainless-steel isolators at two piglets per unit with *ad libitum* access to sterile drinking water and commercial piglet feed. Animal experimental procedures were approved by the Laboratory Animal Welfare and Ethics Committee of the Changchun Veterinary Research Institute (IACUC ofAMMS-11-2022-003).

### Construction of recombinant *L. plantarum*

The truncated S1 domain of PEDV (residues 233-742, GenBank:AKN45969.1) was selected (30), and the endogenous signal peptide 1320 (GenBank:CP016270.1) was linked to its 5′ terminus, while a dendritic cell-targeting peptide (DCpep) and an HA-tag were added to the 3′ terminus. The gene was codon-optimized for *L. plantarum* (LP12) and synthesized as 1320-PEDV tS1-DCpep (31). This fragment was amplified using primers F01 and R01 (Table 1), cloned into the pSIP411 vector via seamless cloning, and electro-transformed into *Lactococcus lactis* NZ3900. Positive colonies were selected on GM17 agar (Haibo, China) containing erythromycin and sequenced for verification. The confirmed plasmid was then transferred into LP12, and positive colonies were screened on MRS agar (Haibo, China) with erythromycin and validated by PCR using the primers 411-test-F and 411-test-R (Table 1). The resulting strain was named as LP12:PEDV tS1. A control strain, LP12:Vector, was constructed using the same method as for the empty pSIP411 vector.

**TABLE 1:** Primers used in this study

Primer	Sequence (5′−3′)	Production size (bp)
F01	TATTACAAGGAGATTTTAGCCATGGAGATTTTAGCCATG	1,858
R01	GGGGTACCGAATTCCTCGAGTCTAGTTAAGCATAATCTGGAACATCAT	
411-test-F	GCTTCCCACACGCATTTCAG	2,486
411-test-R	ATTCTGCTCCCGCCCTTATG	
PEDV-F	ACTCTTCTAGCTGGTACTGTGGC	300
PEDV-R	ATCCTGCAAAGCTGGAATGGC	

### Identification of proteins expressed by the recombinant *L. plantarum*

Positive colonies were cultured in MRS medium (Haibo, China) with erythromycin (10 μg/mL) at 37°C until reaching an OD of 0.3–0.5 and then induced with SppIP (Genscript Biotech, China, 50 ng/mL) for 8 h (32). After centrifugation at 8,000 ×*g* for 3 min, the bacteria were washed with PBS and homogenized with 0.1 μm glass beads for 20 min, and the supernatant was collected for analysis.

For western blotting, 100 μL of protein was mixed with 5× Loading Buffer, boiled for 7 min, and separated using 10% SDS-PAGE. The membrane was blocked with 5% skim milk, incubated with rabbit anti-HA antibody (1:1,000, Proteintech, USA) for 2 h, washed, and incubated with HRP-labeled goat anti-rabbit secondary antibody (1:5,000, Beyotime Biotechnology, China) for 40 min. Detection was performed using ECL (Thermo Fisher, USA).

For immunofluorescence assay (IFA) and flow cytometry (FCM), the induced bacteria were washed and incubated with rabbit anti-HA antibody (1:100, Proteintech, USA) overnight and then with FITC-labeled secondary antibody (1:5,000, Beyotime Biotechnology, China) for 2 h. Fluorescence microscopy and flow cytometry were used to confirm the expression of foreign genes and to quantify the positive expression rates.

### Optimization of expression

To determine the optimal expression conditions of LP12:PEDV tS1, a series of expression parameters were systematically optimized. For the optimization of inducer concentration, LP12:PEDV tS1 was induced with SppIP at concentrations of 50, 100, 150, and 200 ng/mL for 6 h at 37°C. To optimize the induction time, LP12:PEDV tS1 was induced with 100 ng/mL of SppIP at 37°C for 4, 6, 8, 10, 12, 16, 20, and 24 h, respectively. For temperature optimization, LP12:PEDV tS1 was induced with 100 ng/mL of SppIP for 6 h at 33°C, 37°C, and 42°C. Protein samples were prepared according to the methods described above. The samples were quantified using a BCA protein analysis kit, and the expression levels of PEDV tS1 protein in the samples were detected by western blotting.

### Immunization of mice

Thirty mice were randomly divided into three groups: PBS group (*n*=10), LP12:Vector group (*n*=10), and LP12:PEDV tS1 group (*n*=10). The immunization procedure is shown in Fig. 4A. LP12:PEDV tS1 and LP12:Vector were prepared under optimized conditions, and the recombinant bacteria were placed in PBS. After anesthesia, each mouse was inoculated with 20 μL of PBS (negative control) or PBS containing 1×10^9^ CFU of LP12:Vector or LP12:PEDV tS1 via nasal immunization. Every mouse was immunized on days 0 and 14 for consecutive 3 days each time. Samples were collected on days 14, 21, and 28 for detection.

### Immunization of piglet

A total of 10 PEDV-seronegative, 25-day-old Large White piglets were housed in stainless-steel isolators (two per unit) with *ad libitum* access to sterile drinking water and commercial piglet feed. Following a 7-day environmental acclimation period, all piglets were immunized with LP12:PEDV tS1 according to the experimental protocol (Fig. 6A).

Piglets were randomly divided into the LP12:Vector group (*n*=5) and the LP12:PEDV tS1 group (*n*=5). The immunization program is shown in Fig. 5A. LP12:PEDV tS1 and LP12:Vector were prepared under optimized conditions. The bacteria were suspended in PBS at a concentration of 1×10⁹ CFU/mL, and the piglets were inoculated with the suspension through the nose for 3 consecutive days (1, 2, and 3 days). After 14 days, the piglets were inoculated with the suspension in the same manner for 3 consecutive days (14, 15, and 16 days). Body temperature and body weight were recorded every 2 days; peripheral blood, nasopharyngeal lavage fluid (NLF), and fecal samples were collected at 7, 14, 21, 26, and 31 days post-treatment.

### Sample collection

Fecal, bronchoalveolar lavage fluid (BALF), and nasal lavage fluid (NLF) samples were collected from mice and/or piglets, resuspended in PBS with Bovine Serum Albumin (BSA, Sigma-Aldrich, USA) and PMSF (1 mM, Beyotime Biotechnology, China), homogenized, and centrifuged. Supernatants were tested or stored in a −80°C refrigerator until use.

Mesenteric lymph nodes (MLN) were excised postmortem, and cells were isolated by grinding through a 70 μm filter. The cells were washed three times with PBS and processed for further analysis.

### Detection of PEDV and its antibodies in piglets before immunization

Piglet blood was collected before immunization. RNA from samples containing anticoagulants was extracted for RT-PCR detection using primers PEDV-F and PEDV-R (Table 1). Serum from samples containing coagulant was collected by centrifugation, specific antibodies were detected by the indirect enzyme-linked immunosorbent assay (ELISA) method established above after optimization, and PEDV-negative piglets were screened.

### Enzyme-linked immunosorbent assay

The plates were prepared in our laboratory. The wells were coated with 5 μg of PEDV receptor binding domain (RBD) protein and incubated at 4℃ for 12 h. Then, they were sealed with 5% skim milk at 37℃ for 1 h. After cleaning the wells five times with PBS + Tween20 (0.1%) (PBST), serum samples from piglets were prepared at a 1:100 dilution, fecal samples were tested without dilution, and BALF and NLF samples were diluted with PBS at a 1:10 ratio. The plates were then incubated at 37℃ for 1 h. After incubation, the plates were washed five times with 200 μL PBST per well. The secondary antibodies used were goat anti-rat IgA (Abcam) and goat anti-pig IgA (Invitrogen). They were added at a volume of 100 μL per well (diluted 1:10,000) and incubated at 37℃ for 40 min. The plates were then washed five times with 200 μL TBST per well. After washing, the plates were visualized using tetramethylbenzidine (TMB) in the dark for 10 min. Finally, the reaction was stopped by adding 50 μL of 2 M H_2_SO_4_ solution per well, and the absorbance was measured at 450 nm using a microplate reader (Tecan, Switzerland) at a reference wavelength of 630 nm.

### Flow cytometry procedures of cell subsets in MLN

The prepared MLN single-cell suspension was counted, and 100 μL of cells (1×10^6^ cells) was placed in a 1.5 mL centrifuge tube. Blank controls and single-stained samples were also prepared. The sampleswere stained with FITC anti-mouse CD80 (clone 16-10A1), PE/cyanine5 anti-mouse CD86 (clone GL-1), and PE anti-mouse CD11c (clone N41δ) (both from BioLegend, USA) antibodies. Diluted antibodies (10 μL each) were added to the respective sample groups, mixed thoroughly, and incubated at 4°C in the dark for 30 min. Subsequently, the samples were washed twice with PBS and resuspended in 500 μL PBS. The suspension was then passed through a 0.22 μm filter and analyzed using appropriate equipment.

### Antibody neutralization assay

Neutralization antibodies were detected in the serum and feces of mice, as well as in the serum, feces, and NLF of piglets by a virus neutralization assay. The samples were filtered through a 0.22 μm filter screen and irradiated with ultraviolet light for 40 min. Serum, feces, and NLF were inactivated at 56℃ for 30 min, and the inactivated samples were twofold diluted to 50 μL per well in DMEM from 1:2 to 1:256. At the same time, the plates were added 50 μL with 200 TCID_50_ PEDV and mixed at 37℃, 5%CO_2_ incubator for 1 h. Viral cytopathic effects from vero cells upon adding the mixture were observed at 72 h post-incubation at 37℃ in 5%CO_2_ incubator.

### Virus challenge of piglets with PEDV

Nineteen 3-day-old piglets were divided into four groups: mock (*n*=4), PEDV (*n*=5), LP12:Vector (*n*=5), and LP12:S1 (*n*=5). After 3 days of adaptation, nasal administration of LP12:Vector or tS1 was performed daily for the next 5 days. Then, we fed PEDV with a viral titer of 5×10^6^ copies/mL to simulate piglet infection. The survival rate, weight change, and virus shedding were used to evaluate the antiviral effect of LP12:Vector or tS1. The viral copies from the tissue samples, including the duodenum, jejunum, ileum, and colon, were detected by RT-qPCR.

### Statistical tests

Statistical significance was determined by one-way or two-way analyses of variance with Tukey’s or Bonferroni’s multiple comparison test using GraphPad Prism (GraphPad Software, USA). The results are presented as the mean ± SEM. Statistical significance is shown as follows:*P < 0.05, **P < 0.01, ***P < 0.001, and ****P < 0.0001.

## RESULTS

### Construction of recombinant *L. plantarum* expressing PEDV tS1 protein

**Fig 1:**
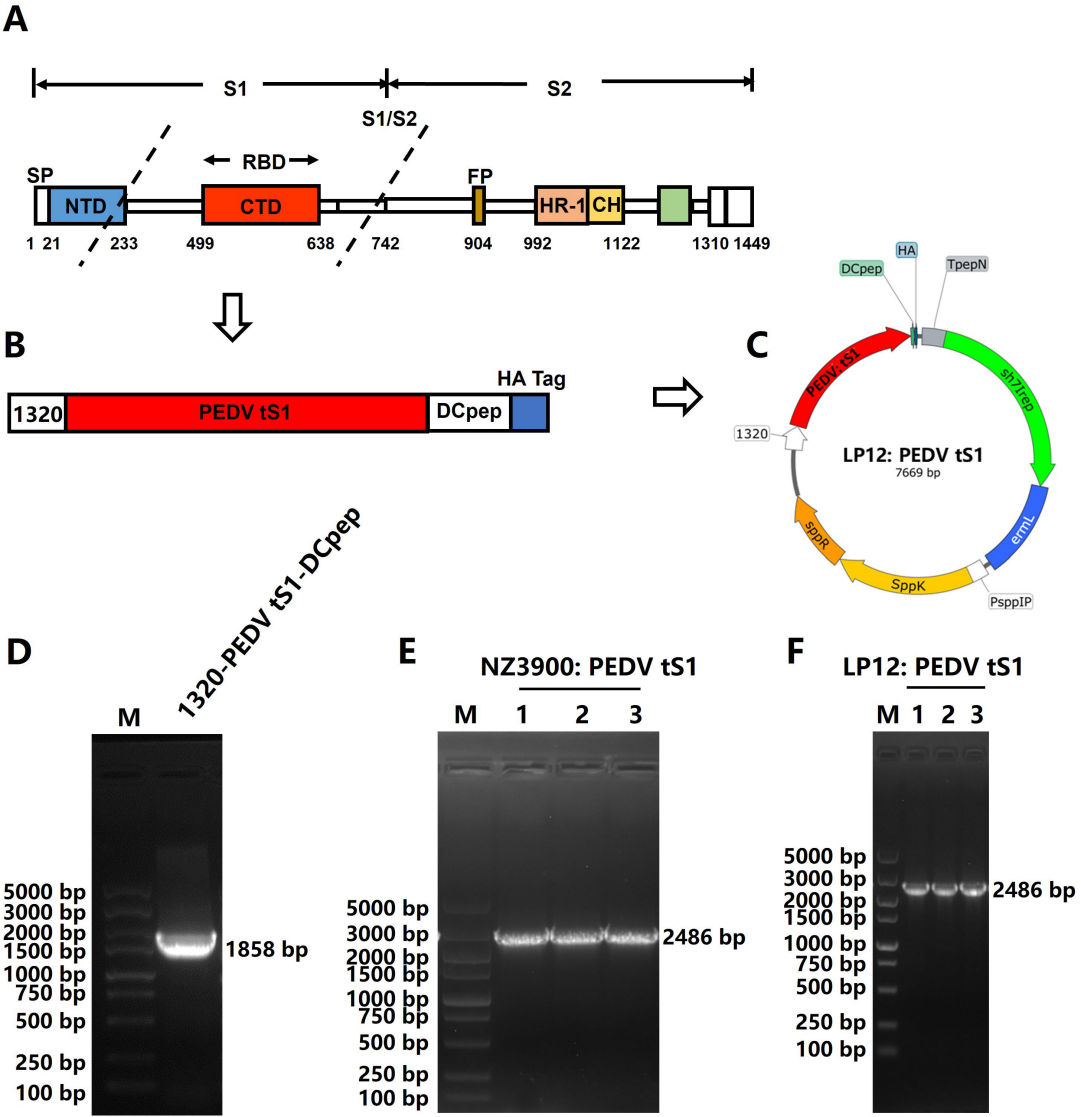
Construction of the recombinant *L.plantarum* strain expressing the PEDV tS1 protein. (**A**)The truncated S1 protein of PEDV encompasses 509 amino acids, spanning from the end of the N-terminal domain (NTD) at amino acid position 233 to amino acid position 742. SS, signal peptide sequence; NTD, N-terminus domain; S1-CTD, S1-C-N-terminus domain; FP, fusion peptide; HR1, heptad repeat 1; HR2, heptad repeat 2. (**B**)Schematic diagram of the inserted sequence, 1320-PEDV tS1-DCpep. 1320, signal peptide 1320; DCpep, DC-targeting peptide; HA, HA-epitope tag. (**C**)Schematic diagram of the recombinant plasmid LP12:PEDV tS1. (**D**)The product of the 1320-PEDV tS1-DCpep gene amplification was 1,858 bp. (**E**)Positive colony PCR product from recombinant *Lactococcus lactis* NZ3900 was 2,486 bp using 411-test-F/R primers. (**F**)Positive colonies from recombinant *L.plantarum* were verified by PCR using the 411-test-F/R primers.

Based on the antigenic profile of the PEDV S protein, the structural domain S1 was selected (33). A 1,320 signal peptide was incorporated at the N-terminus, whereas DCpep and HA tag sequences were appended at the C-terminus to obtain the recombinant sequence PEDV tS1 (Fig. 1). Using agarose gel electrophoresis (AGE), a specific fragment of 1,858 bp was obtained by PCR amplification from the plasmid containing PEDV tS1, which was consistent with the expected size (Fig. 1D). The precise target gene fragment incorporated into the pSIP411 vector by seamless cloning was electrically transformed into *Lactococcus lactis* NZ3900. The recombinant plasmid was extracted and verified by PCR and sequencing using primer 411-test-F/R(Fig.1). Subsequent electro-transformation into LP12 and PCR verification showed a specific band at 2,486 bp (Fig. 1F), which was consistent with the expected result. Sequencing analysis confirmed successful acquisition of the recombinant PEDV tS1 sequence without any nucleotide mutations.

### Identification of tS1 protein expression on LP12:PEDV tS1

**Fig 2:**
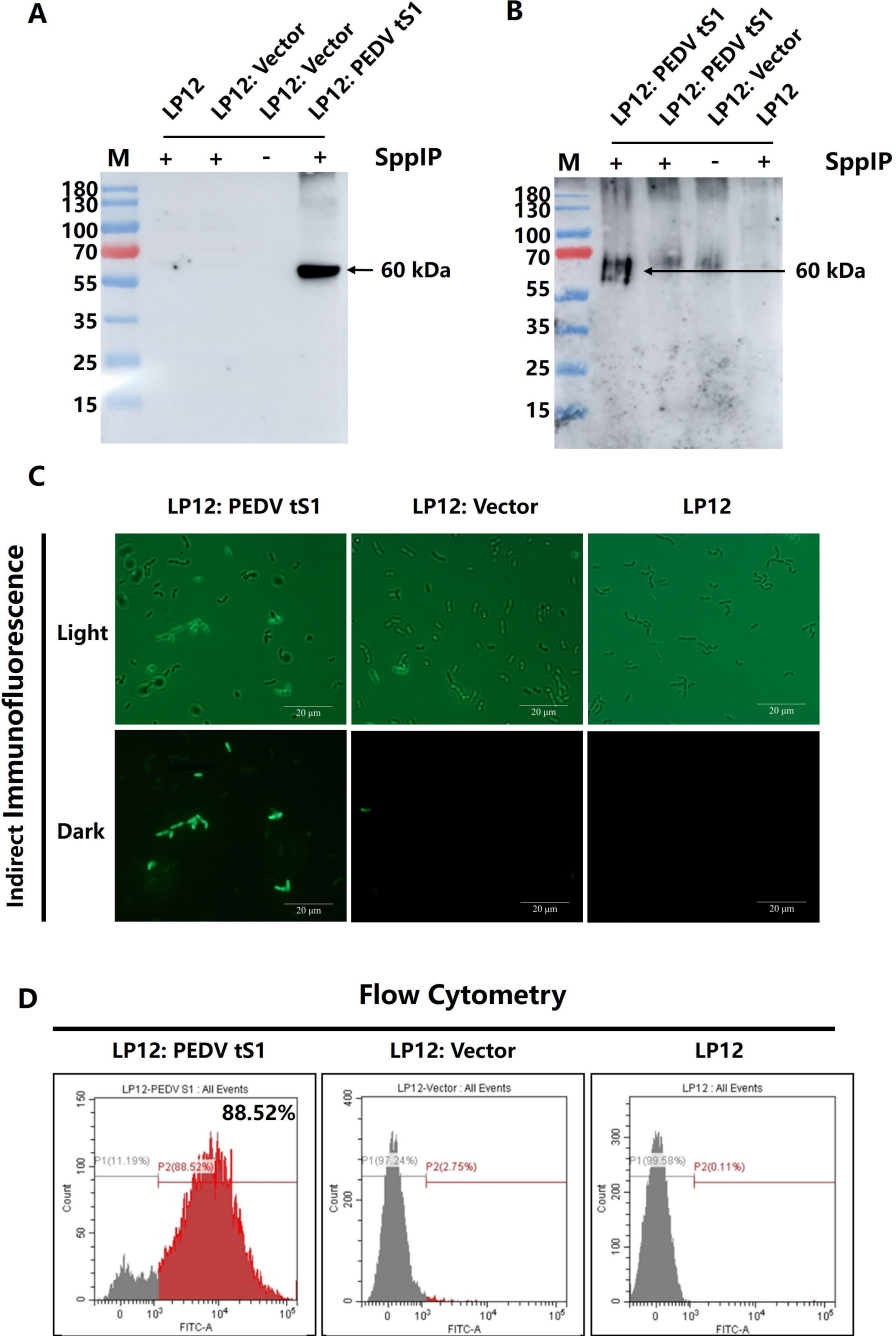
Identification of LP12:PEDV tS1 expressing PEDV tS1 protein. The PEDV tS1 protein expression of LP12:PEDV tS1 was confirmed by western blotting using anti-HA tag rabbit polyclonal antibody (**A**)and anti-PEDV RBD mouse monoclonal antibody(**B**) as primary antibodies. LP12/LP12:Vector/LP12:PEDV tS1 strains were separately induced with(+) or without(−) SppIP. (**C**)The results of the indirect immunofluorescence assay were imaged under a 100× objectivelens of a fluorescence microscope using an excitation wavelength of 488 nm and emission wavelength of 590 nm. (**D**)The display of S1 on the surface of LP12:PEDV tS1 was evaluated using FCM. The induced LP12:Vector and LP12 were used as controls.

To verify the expression of the target protein, western blot analysis was performed using the engineered LP12:PEDV tS1 strain. A distinct band of approximately 60 kDa, corresponding to the tS1 protein, was detected using an anti-HA-tag rabbit polyclonal antibody (1:1,000 dilution, Proteintech, USA) (Fig. 2A) and an anti-PEDVreceptor-binding domain (RBD) mouse monoclonal antibody (1:500 dilution, prepared in-house) (Fig. 2B). To further confirm the surface localization of the PEDV tS1 protein on LP12, the IFA and FCM were performed. The IFA results demonstrated the presence of green fluorescence, indicating successful surface display of the PEDV tS1 protein on LP12 (Fig. 2C). FCM analysis revealed a distinct rightward shift in the fluorescence peak of LP12:PEDV tS1 compared with that of the negative control, with a positive rate of 88.52%(Fig. 2D). In contrast, the LP12:Vector and LP12 control groups showed no significant fluorescence shift. These findings collectively demonstrated that the PEDV tS1 protein was effectively anchored on the surface of LP12, confirming the successful surface display of the recombinant protein.

### Optimization of tS1 protein expression

**Fig 3:**
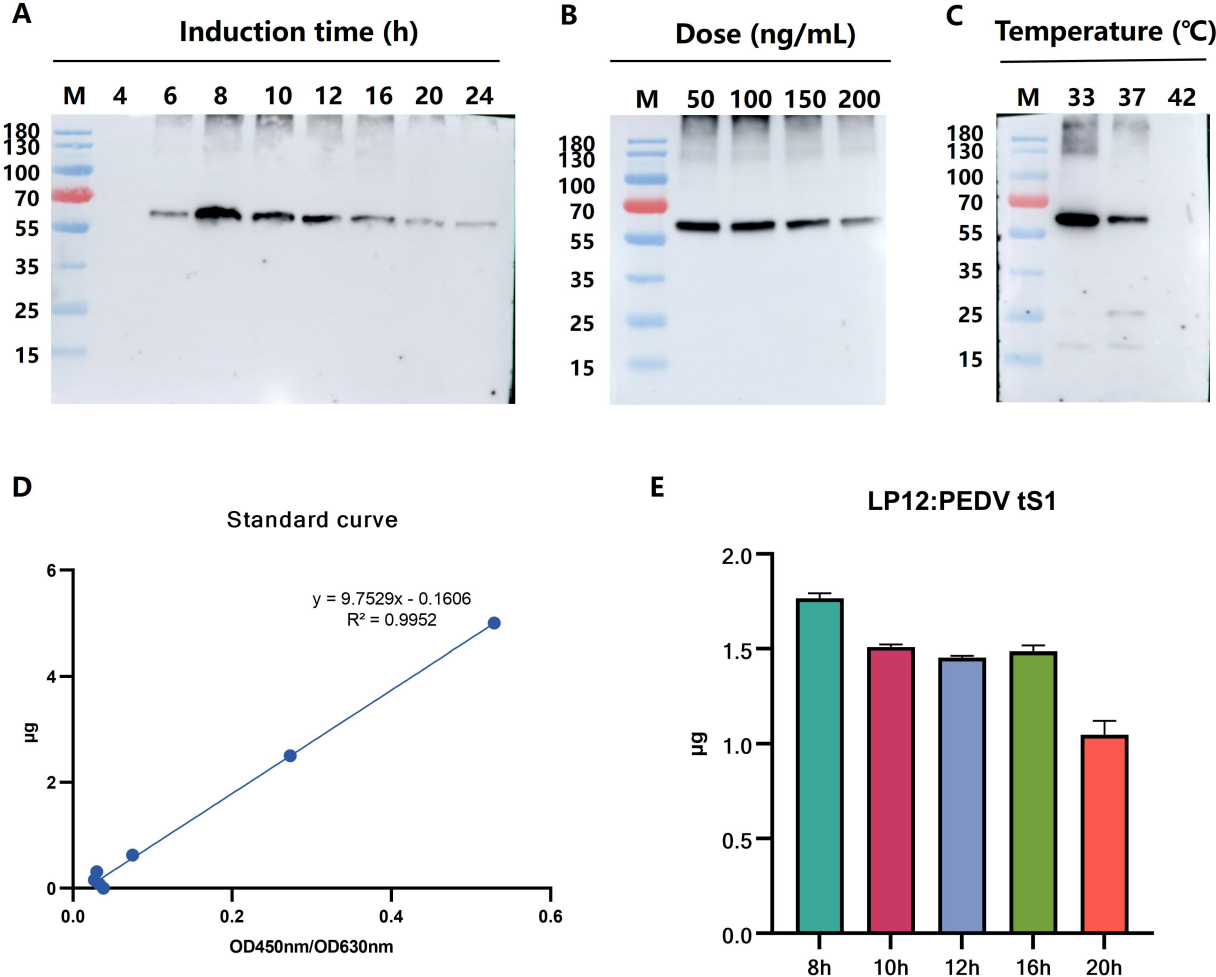
Recombinant protein expression optimization and quantification. The recombinant protein S1 was induced at 4–24 h (2 or 4-h interval) (**A**),50/100/150/200 ng/mL SppIP (**B**),and various incubation temperatures (33°C, 37°C, 42°C) (**C**)and evaluated by western blotting. (**D**)The standard curve of purified PEDV protein was established using ELISA, and the regression equation was obtained as *y*=9.7529*x*−0.1606. (**E**)The level of the target protein was calculated using the equation at 8/10/12/16/20 h post-induction.

The expression level of the recombinant protein was influenced by several factors, including the induction time, inducer concentration, and induction temperature (Fig. 3). To optimize the production of LP12:PEDV tS1 protein, these parameters were systematically investigated. Optimization of the induction time revealed that the highest protein expression level was achieved at 8 h post-induction (Fig. 3A). Similarly, the evaluation of the inducer concentration demonstrated that maximal expression occurred at an inducer concentration of 50 ng/mL (Fig. 3B). Furthermore, temperature optimization experiments indicated that the optimal induction temperature to achieve the highest expression level was 33°C (Fig. 3C). Based on these findings, the optimal conditions for maximizing the expression of LP12:PEDV tS1 were determined to be induction at 33°C with an inducer concentration of 50 ng/mL for a duration of 8 h. These optimized parameters ensured the highest yield of the recombinant protein under the experimental conditions.

### Quantitation of target protein

The expression level of the target protein from LP12:PEDV tS1 was quantified using indirect ELISA. Initially, purified PEDV S protein was quantified to generate a standard curve, with the optical density at 450 nm (OD450) plotted on the *x*-axis and the expression level of the target protein on the *y*-axis (Fig. 3D). A total of 1×10^9^ CFU LP12:PEDV tS1 was induced, yielding 410 μg total protein as determined by BCA assay. By substituting the OD450 values obtained at various time points into the regression equation, the expression level of the target protein 8 h post-induction was calculated to be 0.00176 μg. Using the regression equation, the expression levels of the target protein at different time points (8, 10, 12, 16, and 20 h) were calculated (Fig. 3E) (Table 2). The results demonstrated that the highest expression level of the target protein (1.7656 μg) was achieved at 8 h post-induction. However, as the induction time increased, the expression level gradually decreased, reaching 1.0476 μg at 20 h. These findings indicated a time-dependent decrease in target protein expression following the initial peak at 8 h.

**TABLE 2:** Target protein expression at different time points

Time point	1×10^9^CFU total protein (μg)	Loading quantity (μg)	Amount of target protein calculated by standard curve (μg)	Proportion of expressed proteins to total proteins	Amount of PEDV tS1 expressed at 1×10^9^CFU (μg)
8h	410	0.41	0.00176	0.43%	1.7657
10h	410	0.41	0.00151	0.368%	1.5086
12h	410	0.41	0.00146	0.355%	1.4537
16h	410	0.41	0.00148	0.362%	1.4857
20h	410	0.41	0.00105	0.256%	1.0476

### Antigen-specific IgA induced by recombinant *L.plantarum* in mice

**Fig 4:**
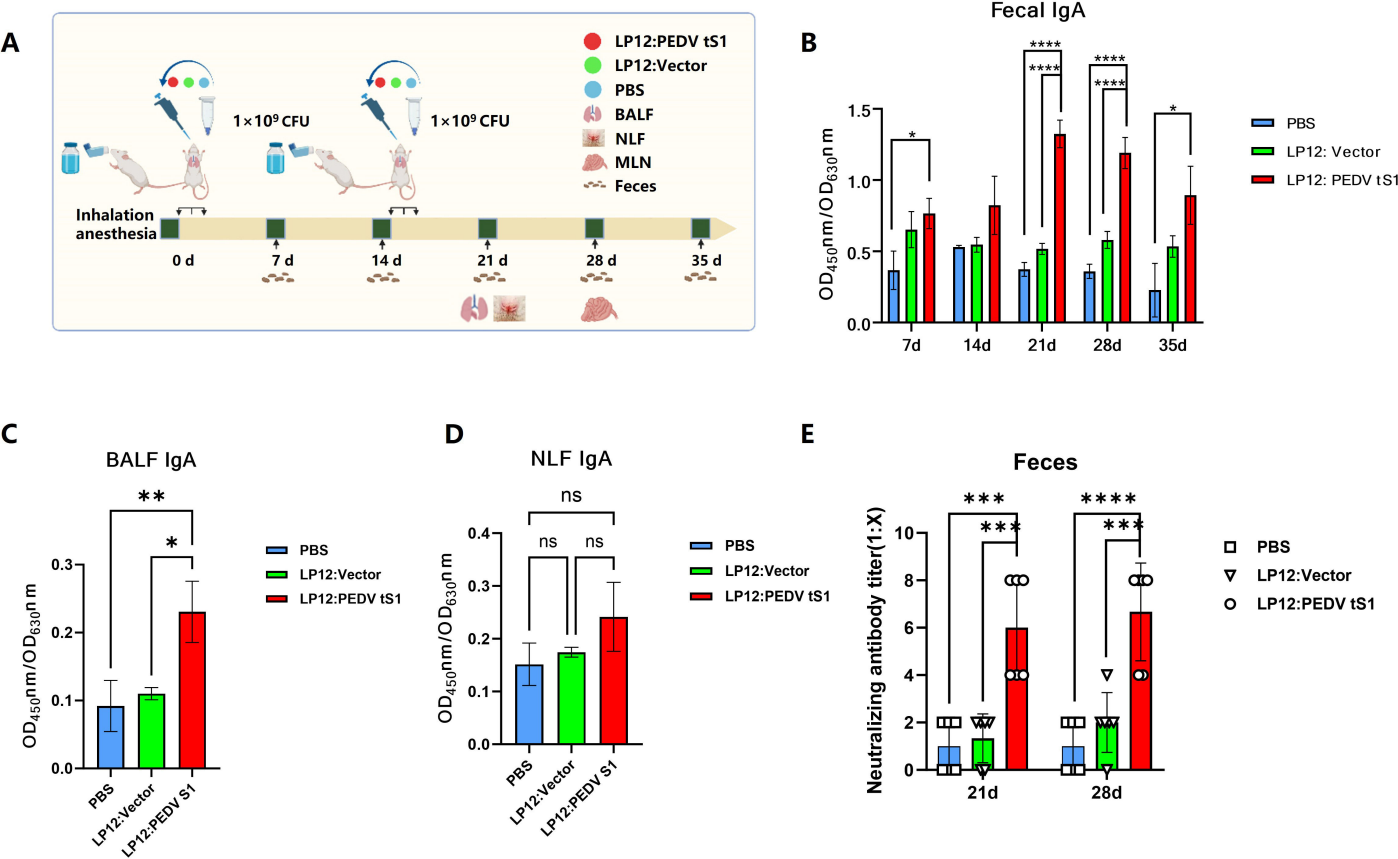
Immunological evaluation of LP12:PEDV tS1 in mice. (**A**)Experimental design and immunization schedule. Three groups of mice were administered PBS, 1×10^9^CFU of LP12:Vector, or LP12:PEDV tS1, respectively, via the intranasal route. The prime/boost immunization was administered by 3 consecutive days with a 2-week interval. Samples were collected on days 7, 14, 21, 28, and 35. The levels of specific sIgA from PBS (blue), LP12:Vector (green), and LP12:PEDV tS1 (red) groups’ feces(**B**), bronchoalveolar lavage fluid (BALF) on day 21(**C**), and nasal lavage fluid (NLF) on day 21(**D**) were detected by ELISA. (**E**)Neutralizing activity of fecal sIgA in immunized mice on day 21 in each group. Statistical significance is denoted as follows: *P< 0.05;**P< 0.01; ***P＜0.001; ****P< 0.0001.

To study the activation effect of LP12:PEDV tS1 on humoral immunity in mice, specific fecal sIgA was detected 7, 14, 21, 28, and 35 days after immunization, and those in BALF and NLF were detected at day 21. The results showed that, compared to the PBS group, specific fecal sIgA levels of LP12:PEDV tS1 began to significantly increase on day 7 (*P* < 0.05) (Fig. 4B). Compared to the PBS and LP12:Vector groups, the specific fecal sIgA levels of LP12:PEDV tS1 increased significantly (*P* < 0.0001) and reached their highest point at 21 days, remaining high at 28 and 35 days. In BALF, the specific sIgA levels in the LP12:PEDV tS1 group were significantly higher than those in the PBS and LP12:Vector groups (*P* < 0.05or*P* < 0.01, respectively) (Fig. 4C). In the NLF, there was an increase in the experimental group compared to that in the control group, but no statistical difference was observed (Fig. 4D). These results indicated that LP12:PEDV tS1 successfully induced specific sIgA in mice via the nasal vaccination route.

### Determination of neutralizing antibody titer

Fecal neutralizing antibody titers from mice were detected using the virus neutralization assay on days 21 and 28, respectively (34, 35). The results showed that, compared to the control groups (PBS and LP12:Vector), the titer level of neutralizing antibodies against PEDV reached 1:8 (*P* < 0.001or*P* < 0.0001) (Fig. 4E). These results indicate that neutralizing antibodies were produced in the feces.

### Activation of dendritic cells (DCs) in MLN by the LP12:PEDV tS1

**Fig 5:**
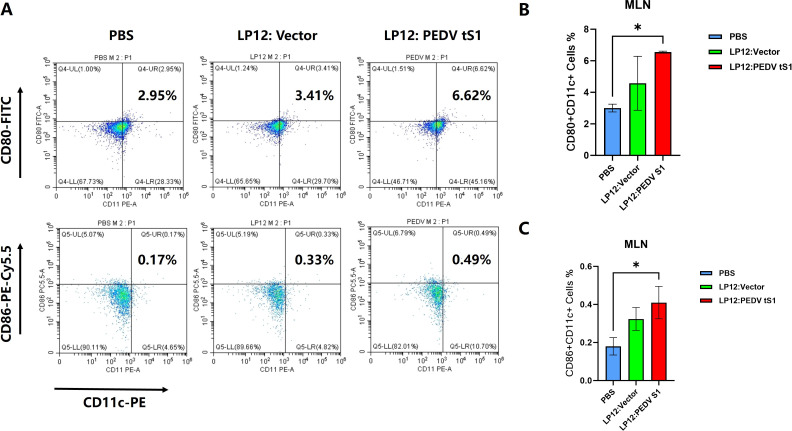
Characterization of mature DC from immunization mice’mesenteric lymph node (MLN) induced by *L. plantarum* expressing PEDV tS1. (**A**)Representative flow cytometry analysis of DCs (MLN) in PBS, LP12:Vector and LP12:PEDV tS1 group. Frequencies of CD11c^+^CD80^+^(**B**)and CD11c^+^CD86^+^ (**C**)dendritic cell subset in response to stimulation with PBS (blue), LP12:Vector (green), and LP12:PEDV tS1 (red) are shown. Statistical significance is denoted as follows: *P< 0.05.

To explore the immunological effect of LP12:PEDV tS1 on DCs activation in the MLN of mice, we detected the expression of the activated markers CD80 and CD86 on the surface of DCs. The DCs’ channel selection method in MLN was shown in figure (Fig. 5A), and the results showed that the frequency of CD11^+^ CD80^+^ subsets in the LP12:PEDV tS1 group was significantly higher than that in the PBS group (*P* < 0.05), while there was no significant difference between the LP12:Vector group and the LP12:PEDV tS1 group (*P* > 0.05) (Fig. 5B). The frequency of the CD11c^+^ CD86^+^ subset in the LP12:PEDV tS1 group was significantly higher than that in the PBS group (*P* < 0.05), and there was no significant difference between the LP12:Vector group and LP12:PEDV tS1 group (*P*>0.05) (Fig. 5C). These results indicate that recombinant *L. plantarum* activated DCs in the MLN of mice.

### Immune responses in piglet induced by recombinant *L. plantarum*

**Fig 6:**
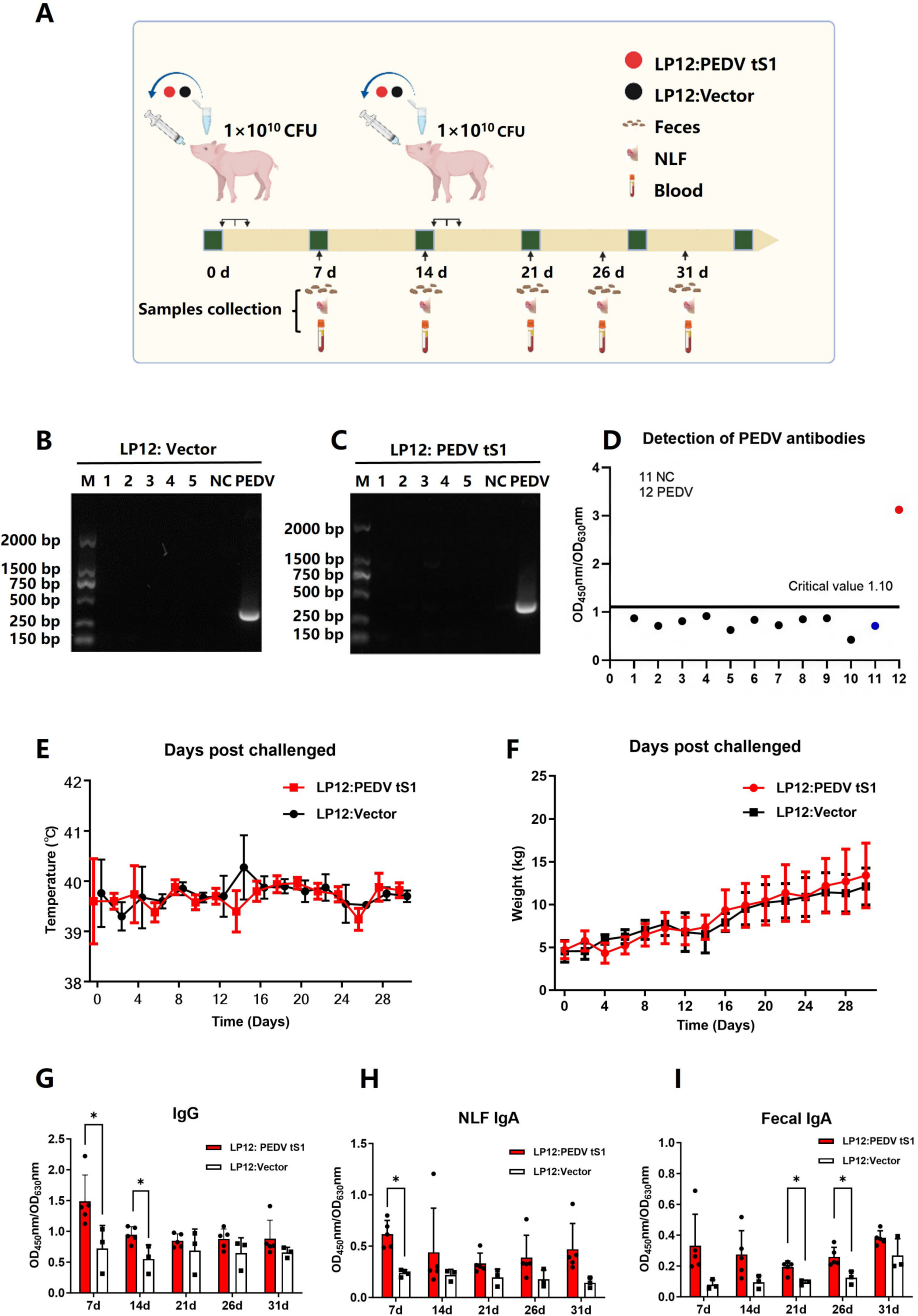
Immunological evaluation of LP12:PEDV tS1 in porcine model. (**A**)Experimental design and immunization schedule. Two groups of piglets were administered 1×10^10^CFU of LP12:Vector or LP12:PEDV tS1, respectively, via the intranasal route. The prime/boost immunization was administered by 3 consecutive days with a 2-week interval. Samples were collected on days 7, 14, 21,26, and 31. (**B-C**)Ten piglets were divided into two groups to detect PEDV gene according to RT-PCR method. (**D**)The serum PEDV antibody was detected by indirect ELISA. The critical value was 1.10. (**E**)Piglettemperature and (**F**)weight change were measured. (**G**)Specific serum IgG, (**H**)specific sIgA in the nasal lavage fluid (NLF), and (**I**)specific fecal sIgA of nasally administrated piglets were compared. Statistical significance is denoted as follows: *P< 0.05.

Using piglets as a model, anti-PEDV-specific sIgA and IgG were detected to evaluate the ability of LP12:PEDV tS1 to induce systemic and mucosal immune responses. Ten piglets were selected and numbered. They were subjected to RT-PCR and indirect ELISA analyses following laboratory-established protocols. The results revealed that the nucleic acids and antibodies in the selected piglets were negative (Fig. 6B through D). The body temperature of the piglets was measured and weighed once every 2 days. Body temperature monitoring data (Fig. 6E) revealed that piglet body temperatures remained within the range of 38.5°C to 39.5°C, with no significant fluctuations associated with immunization. Likewise, body weight measurements (Fig. 6F) demonstrated a steady increase in body weight across all piglet groups. These results showed that the recombinant *L. plantarum* and immunogen did not affect the normal growth of piglets, and the safety was high. At 7 days post-immunization (dpi), serum IgG levels in the immunized group increased significantly (*P* < 0.05). These levels declined at 14 dpi yet remained significantly elevated compared with the LP12:Vector group (*P* < 0.05), while no significant differences in serum IgG levels were observed among groups at 21, 28, and 31 dpi (*P* > 0.05) (Fig. 6G). After LP12:PEDV tS1 nasal drip immunization, the specific sIgA in the NLF of piglets was significantly increased at day 7 (*P* < 0.05), and on days 14, 21, 26, and 32, compared with the control LP12:Vector group, the sIgA of LP12:PEDV tS1 increased, but there was no statistical difference (*P* > 0.05) (Fig. 6H). In addition, sIgA in the feces of piglets showed an increasing trend after immunization, and at days 21 and 26, the LP12:PEDV tS1 group was significantly higher than the LP12:Vector group (*P* < 0.05) (Fig. 6I). In conclusion, systemic and mucosal immunity in piglets were induced by LP12:PEDV tS1 immunization.

The neutralizing capacities of serum antibodies, fecal antibodies, and NLF antibodies in piglets intranasally immunized with recombinant *L. plantarum* against PEDV showed inhibitory activity against viral infection. The average titer of neutralizing antibody in the serum was 1:63, and the most effective titer was 1:81, which was significantly different from that of the control group LP12:Vector (*P* < 0.05) (Fig. S1A). The neutralizing antibody titer in feces was 1:4, which was not significantly different from that in the control group (*P* > 0.05) (Fig. S1B). The neutralizing antibody titer in NLF was 1:4, with no statistically significant difference (*P* > 0.05) (Fig. S1C). Thus, the above results indicate that serum IgG-neutralizing antibodies are produced, whereas sIgA is not. The lack of a sufficient vaccination dose may be the main reason.

### Efficient protection against PEDV challenges by nasal administration of LP12:PEDV tS1

**Fig 7:**
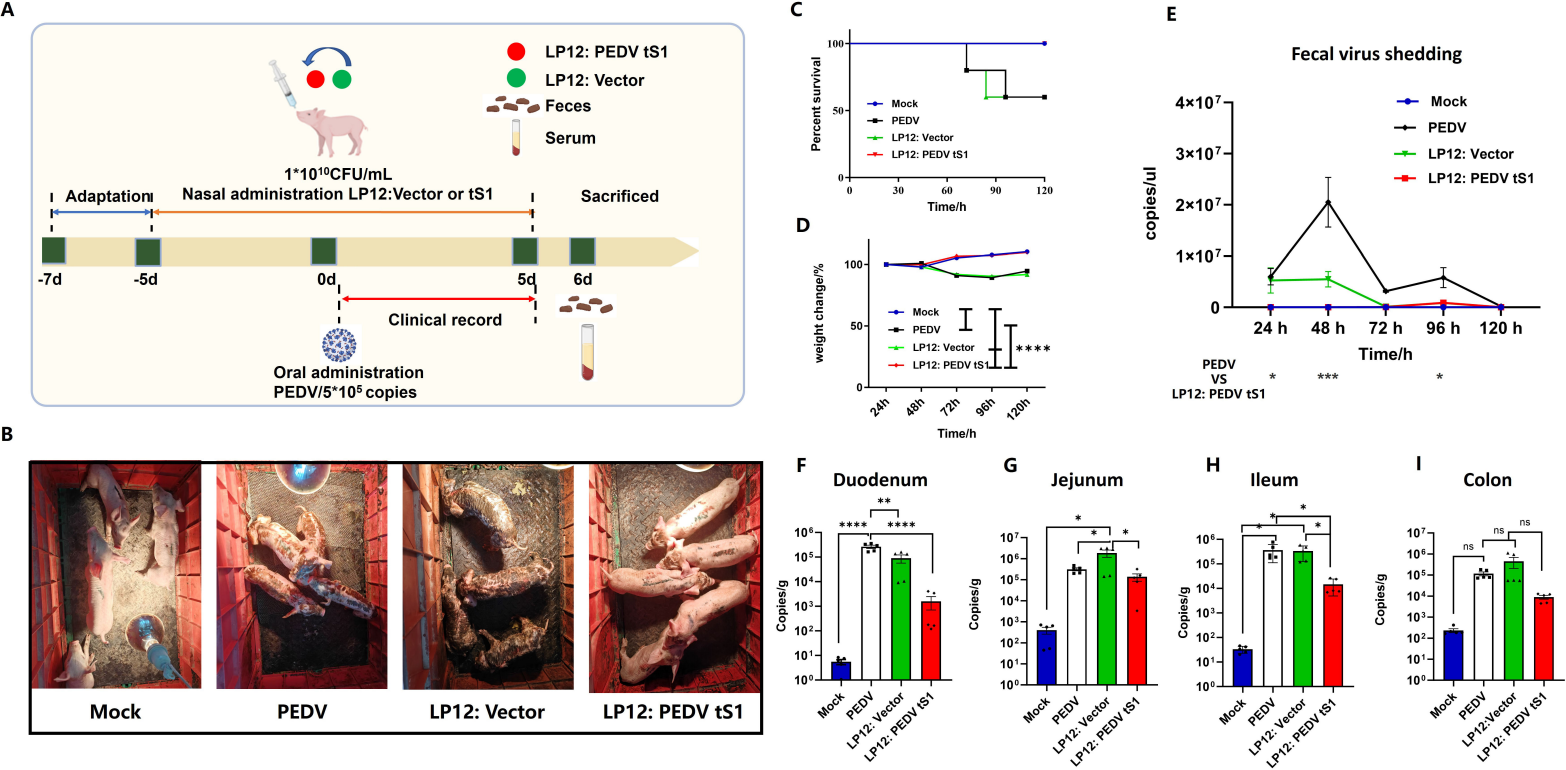
LP12:PEDV tS1 provides an efficient protection against PEDV challenges in a piglet infection model. (**A**)Viral challenges and immunization schedule. Two groups of piglets were administered 1×10^10^CFU of LP12:Vector or LP12:PEDV tS1 for 5 days, respectively, via the intranasal route. Viral challenge with PEDV subsequently occurs in PEDV, LP12:Vector, and LP12:PEDV tS1 groups. Consecutive bacteria nasal administration was sustained for 5 days post-infection. (**B**)Clinical symptoms in Mock (blue), PEDV (white), LP12:Vector, and LP12:PEDV tS1 groups are shown. (**C**)Survival rate, (**D**)weightchange, and (**E**)fecal virus shedding were evaluated. In each group, PEDV titers from duodenum (**F**),jejunum (**G**),ileum (**H**),and colon (**I**)were detected by RT-qPCR.

Although no sharp vaccination data were previously shown in a porcine model, we assessed its efficacy by viral challenge. To enhance antigen exposure, we augmented the frequency of vaccination in the piglets. Notably, piglets immunized with LP12:PEDV tS1 exhibited significantly attenuated clinical manifestations, including reduced diarrhea and dehydration severity, compared with the viral challenge control group (Fig. 7B). The immunization cohort demonstrated a marked improvement in the survival rate (100%) relative to the PEDV/LP12:Vector group (60%) (Fig. 7C). Furthermore, weight gain in immunized piglets paralleled that of the mock group and was substantially greater than that observed in both the PEDV and LP12:Vector groups (Fig. 7D). Fecal virus shedding detected with anal swab samples was significantly diminished by LP12:PEDV tS1 immunization, indicating more powerful virus elimination (Fig. 7E). RT-qPCR analysis of intestinal tissue samples was performed to quantitatively verify viral load reduction. The results revealed statistically significant differences in viral titers across all small intestinal segments between the immunized and PEDV/LP12:Vector group (Fig. 7F through H). A lower viral titer in the colon was observed in the immunized group (Fig. 7I). Collectively, these findings demonstrate that LP12:PEDV tS1 confers rapid protective immunity in piglets.

## DISCUSSION

Zoonotic viruses, including coronaviruses, represent significant public health threats because of their capacity for cross-species transmission (36). PEDV owns several virological characteristics, and its infection in bats and mice has corroborated its potential for cross-species transmission (1, 3, 37, 38). To hedge against potential viral spillover, interspecies variability should be taken into account during vaccine design. However, nucleic acid vaccines often require codon optimization to enhance translational efficiency and immunogenicity of the target host (39). These evidences highlight the need to consider species-specific characteristics during the development of cross-species vaccines. To avoid multiple-codon optimization, we selected a design strategy-based protein subunit principle to solve the problems caused by cross-species vaccination.

Lactic acid bacteria (LAB) have emerged as pivotal platforms for mucosal vaccine delivery owing to their ability to colonize host mucosal surfaces and facilitate antigen presentation, eliciting robust local and systemic immune responses (28, 40–42). By expressing the antigenic proteins themselves, this platform solves the problem of immunization for different species and reduces the cost of the purification process. In this experiment, *Lactiplantibacillus plantarum* strain LP12 was chosen as the vector for the construction and expression of the PEDV tS1 protein. However, heterologous protein expression in LAB is often suboptimal, primarily due to intracellular proteolytic degradation or cytotoxicity-induced bacterial cell death, which collectively impair protein yield. To address these limitations, the 1,320 signal peptide was used to anchor the exogenous protein on the surface of *L. plantarum*, thereby mitigating intracellular degradation and enhancing stability (43). Through codon optimization and expression conditions screening, the expression level of exogenous genes was markedly improved. Consequently, in this study, the target protein expression level in 1×10^9^ CFU of recombinant *L. plantarum* was quantified by ELISA, revealing a yield of 1.7657 μg. Further analysis indicated that the target protein constituted 0.43% of the total protein content at an immunization dose of 1×10^9^ CFU. These results provide a quantitative foundation for subsequent immunization studies in murine and porcine models, underscoring the potential of *L. plantarum* as a mucosal vaccine delivery system.

As the primary interface between the external environment and respiratory system, the respiratory tract serves as the principal portal of entry for numerous respiratory pathogens, including those responsible for influenza and pneumonia (44, 45). Among the respiratory tract structures, the nasal cavity plays a vital role as the first line to defend against the invasion of such microorganisms (46). The anatomical connection of the nasal cavity to the external environment simplifies the targeted delivery of vaccines or therapeutic agents to the nasal mucosal epithelium, which can stimulate M-like cells within the nasal-associated lymphoid tissue (NALT)(47). These specialized cells, analogous to intestinal M cells, exhibit the selective uptake of particulate antigens (48). This process facilitates B cell activation, subsequent entry into the systemic circulation, and migration to mucosal tissues while concurrently promoting the synthesis of secretory immunoglobulin A (sIgA).

Secretory IgA serves as a primary immunological defense mechanism by binding to and neutralizing pathogenic agents or toxins present in mucosal secretions (49). In the current study, specific sIgA antibodies—the key effector molecule of mucosal immunity—were detected in murine fecal samples, bronchoalveolar lavage fluid, and porcine fecal and nasal lavage specimens, highlighting the pivotal potential of recombinant *L.plantarum* in antiviral defense. Following intranasal immunization, neutralizing antibodies were identified in murine fecal matter, porcine serum, and feces, with significant titer elevations observed in porcine blood samples, indicative of the viral neutralization capacity.

Nasal vaccination strategies in swine have demonstrated efficacy in controlling outbreaks of intestinal mucosal viral infections, including those caused by rotaviruses (50), pseudorabies viruses (51), and porcine reproductive and respiratory syndrome viruses (52). Unlike oral delivery of PEDV vaccines based *L.plantarum* vector, intranasal immunization avoids gastric degradation, preserves the antigen activity, rapidly induces intestinal mucosal immunity via the nasal-associated lymphoid tissue–common mucosal immune system (NALT–CMIS) axis, and facilitates piglet handling (53). Mesenteric lymph nodes (MLNs), as a core hub of the intestinal mucosal immune system, are pivotal for antigen capture, presentation, and immune cell activation. Upon intranasal immunization, *Lactobacillus plantarum* traverses the nasal epithelial barrier via intrinsic mucosal adhesion—its surface lipoteichoic acid and adhesins bind to nasal epithelial mucins and integrins, evading mucosal clearance (54). The cross-mucosal immune network between nasal-associated lymphoid tissue (NALT) and gut-associated lymphoid tissue (GALT) enables NALT-resident immature DCs to capture recombinant *L. plantarum*, which migrates to MLNs via lymphatic circulation, laying the antigenic foundation for DC responses (55).

The specific immune response involves a complex interplay between multiple cell types, including B lymphocytes, T lymphocytes, and dendritic cells (DCs). CD80 and CD86, key co-stimulatory molecules that interact with T cells, enhance cytokine secretion (e.g., IL-2) and prolong T cell activity. Upon activation of B cells, monocytes, and dendritic cells, both CD80 and CD86 are significantly upregulated (56). Previous research has demonstrated that recombinant *L. plantarum* containing DCpep can effectively activate intestinal dendritic cells (DCs) in mice, followed by gradual activation of DCs in Peyer’s patches and MLNs (57). In this study, our results showed significant upregulation of CD80 and CD86 in the MLNs of mice immunized with LP12:PEDV tS1 after 21 days compared to the control group (PBS group). LP12:Vector group demonstrated moderate upregulation although the difference was not statistically significant. These findings suggest that LP12:PEDV tS1 may activate B cell populations and ripe the dendritic cells, strengthening their capacity to activate T lymphocytes. This facilitates the recognition of antigens by T lymphocytes and the subsequent immune responses. The LP12:Vector group also exhibited increased antigen presentation capability, indicating their potential as adjuvants to enhance the immune response of the host and provide certain protective effects.

Importantly, we assessed the potential of LP12:PEDV tS1 through a viral challenge and confirmed its efficacy in a newly established piglet infection model. Strikingly, nasal immunization with recombinant *L. plantarum* LP12:PEDV tS1 conferred rapid protection against PEDV following multiple administration, demonstrating both safety and efficacy *in vivo*. Therefore, nasal administration of recombinant *L. plantarum* LP12:PEDV tS1 may be a promising strategy for eliciting mucosal immune responses. This study has taken a progressing step in the development of a mucosal vaccine against PEDV using a lactic acid bacterial delivery vector. Although limited by relatively low antigen expression levels, this study establishes a noninvasive, cross-species immunization strategy and demonstrates its efficacy through well-validated piglet challenge models that assess both protective immunity and safety.

### Conclusion

In conclusion, recombinant *L. plantarum* LP12:PEDV tS1 was successfully constructed as a mucosal vaccine candidate. Truncated S1 protein was efficiently displayed on the surface of the bacteria and revealed good antigenicity. Surface display of the PEDV tS1 antigen on LP12 can regulate the activation status of immune cells, improve DC function, and enhance T-cell responses. We also demonstrated that LP12:PEDV tS1 enhances specific sIgA antibody levels in mouse feces and bronchoalveolar lavage fluid, as well as in piglet feces, indicating the induction of mucosal immune responses. Furthermore, it effectively and quickly induced an antiviral response against PEDV infection in piglets. This study highlights the potential of LP12 expressing the PEDV tS1 protein as a candidate mucosal vaccine because it demonstrates an effective immune response against PEDV.

## SUPPLEMENTAL MATERIAL

Fig. S1 aem.00386-26-s0001.docx Virus neutralizing activity of serum, feces, and NLF.

ASM does not own the copyrights to Supplemental Material that may be linked to, or accessed through, an article. The authors have granted ASM a non-exclusive, world-wide license to publish the Supplemental Material files. Please contact the corresponding author directly for reuse.

## References

[1] Chen J, Wang Z, Lin S, Gao M, Shao Y, Li S, Chen Q, Cui Y, Hu Y, Liu G. 2025. Insights into cross-species infection: porcine epidemic diarrhea virus infections in the rodent. Virol Sin 40:301–313. doi:10.1016/j.virs.2025.03.012 40157605 PMC12282439

[2] Mao L, Cai X, Li J, Li X, Li S, Li W, Lu H, Dong Y, Zhai J, Xu X, Li B. 2025. Discovery of a novel betacoronavirus 1, cpCoV, in goats in China: the new risk of cross-species transmission. PLoS Pathog 21:e1012974. doi:10.1371/journal.ppat.1012974 40100842 PMC11918373

[3] Li J, Tian F, Xiang R, Zhang S, Ma S, Zhao Y, Que T, Li S, Sothea Y, Li J, Jiang J, Tong Y. 2026. One Health surveillance of bat virome in mainland Southeast Asia and adjacent regions. hLife 4:107–122. doi:10.1016/j.hlife.2025.10.008

[4] Maclachlan NJ, Dubovi EJ, Barthold SW, Swayne DE, Winton JR. 2017. Fenner’s veterinary virology. Elsevier.

[5] Yang D-K, Kim H-H, Lee S-H, Yoon S-S, Park J-W, Cho I-S. 2018. Isolation and characterization of a new porcine epidemic diarrhea virus variant that occurred in Korea in 2014. J Vet Sci 19:71–78. doi:10.4142/jvs.2018.19.1.71 28693308 PMC5799402

[6] Van Diep N, Sueyoshi M, Norimine J, Hirai T, Myint O, Teh APP, Izzati UZ, Fuke N, Yamaguchi R. 2018. Molecular characterization of US-like and Asian non-S INDEL strains of porcine epidemic diarrhea virus (PEDV) that circulated in Japan during 2013-2016 and PEDVs collected from recurrent outbreaks. BMC Vet Res 14:96. doi:10.1186/s12917-018-1409-0 29540176 PMC5852955

[7] Kocherhans R, Bridgen A, Ackermann M, Tobler K. 2001. Completion of the porcine epidemic diarrhoea coronavirus (PEDV) genome sequence. Virus Genes 23:137–144. doi:10.1023/a:1011831902219 11724265 PMC7089135

[8] Lin C-M, Saif LJ, Marthaler D, Wang Q. 2016. Evolution, antigenicity and pathogenicity of global porcine epidemic diarrhea virus strains. Virus Res 226:20–39. doi:10.1016/j.virusres.2016.05.023 27288724 PMC7111424

[9] Duarte M, Tobler K, Bridgen A, Rasschaert D, Ackermann M, Laude H. 1994. Sequence analysis of the porcine epidemic diarrhea virus genome between the nucleocapsid and spike protein genes reveals a polymorphic ORF. Virology (Auckl) 198:466–476. doi:10.1006/viro.1994.1058 PMC7131309 8291230

[10] Li W, van Kuppeveld FJM, He Q, Rottier PJM, Bosch B-J. 2016. Cellular entry of the porcine epidemic diarrhea virus. Virus Res 226:117–127. doi:10.1016/j.virusres.2016.05.031 27317167 PMC7114534

[11] Hou Y, Wang Q. 2019. Emerging highly virulent porcine epidemic diarrhea virus: molecular mechanisms of attenuation and rational design of live attenuated vaccines. Int J Mol Sci 20:5478. doi:10.3390/ijms20215478 31689903 PMC6862049

[12] Li J, Li Y, Liu P, Wang X, Ma Y, Zhong Q, Yang Q. 2022. Porcine epidemic diarrhea virus infection disrupts the nasal endothelial barrier to favor viral dissemination. J Virol 96:e00380-22. doi:10.1128/jvi.00380-22 35435723 PMC9093128

[13] Yuan C, Zhang P, Liu P, Li Y, Li J, Zhang E, Jin Y, Yang Q. 2022. A novel pathway for porcine epidemic diarrhea virus transmission from sows to neonatal piglets mediated by colostrum. J Virol 96:e00477-22. doi:10.1128/jvi.00477-22 35758666 PMC9327711

[14] Maltseva M, Galipeau Y, Renner TM, Deschatelets L, Durocher Y, Akache B, Langlois M-A. 2022. Characterization of systemic and mucosal humoral immune responses to an adjuvanted intranasal SARS-CoV-2 protein subunit vaccine candidate in mice. Vaccines 11:30. doi:10.3390/vaccines11010030 36679875 PMC9865305

[15] Sandbrink JB, Koblentz GD. 2022. Biosecurity risks associated with vaccine platform technologies. Vaccine 40:2514–2523. doi:10.1016/j.vaccine.2021.02.023 33640142 PMC7904460

[16] Kim H, Kirtane AR, Kim NY, Rajesh NU, Tang C, Ishida K, Hayward AM, Langer R, Traverso G. 2023. Gastrointestinal delivery of an mRNA vaccine using immunostimulatory polymeric nanoparticles. AAPS J 25:81. doi:10.1208/s12248-023-00844-z 37589795 PMC10845796

[17] Plotkin SA, Plotkin SL. 2011. The development of vaccines: how the past led to the future. Nat Rev Microbiol 9:889–893. doi:10.1038/nrmicro2668 21963800

[18] Kis Z, Shattock R, Shah N, Kontoravdi C. 2019. Emerging technologies for low-cost, rapid vaccine manufacture. Biotechnol J 14:e1800376. doi:10.1002/biot.201800376 31286673

[19] Graham BS. 2020. Rapid COVID-19 vaccine development. Science 368:945–946. doi:10.1126/science.abb8923 32385100

[20] Su K, Wang Y, Yuan C, Zhang Y, Li Y, Li T, Song Q. 2023. Intranasally inoculated bacterium-like particles displaying porcine epidemic diarrhea virus S1 protein induced intestinal mucosal immune response in mice. Front Immunol 14:1269409. doi:10.3389/fimmu.2023.1269409 37790942 PMC10544335

[21] Bae J-L, Lee J-G, Kang T-J, Jang H-S, Jang Y-S, Yang M-S. 2003. Induction of antigen-specific systemic and mucosal immune responses by feeding animals transgenic plants expressing the antigen. Vaccine 21:4052–4058. doi:10.1016/s0264-410x(03)00360-8 12922142

[22] Li M, Sun X, Chen Y, Wang S, Li Q, Wang Y, Wang Y, Li R, Ding P, Zhang G. 2024. Enhancing humoral and mucosal immune response of PED vaccine candidate by fusing S1 protein to nanoparticle multimerization. Vet Microbiol 290:110003. doi:10.1016/j.vetmic.2024.110003 38262114

[23] Hu Z, Tian X, Lai R, Ji C, Li X. 2023. Airborne transmission of common swine viruses. Porc Health Manag 9:50. doi:10.1186/s40813-023-00346-6 PMC10619269 37908005

[24] Li Y, Wu Q, Huang L, Yuan C, Wang J, Yang Q. 2018. An alternative pathway of enteric PEDV dissemination from nasal cavity to intestinal mucosa in swine. Nat Commun 9:3811. doi:10.1038/s41467-018-06056-w 30232333 PMC6145876

[25] Yuan C, Jin Y, Li Y, Zhang E, Zhang P, Yang Q. 2021. PEDV infection in neonatal piglets through the nasal cavity is mediated by subepithelial CD3^+^ T cells. Vet Res 52:26. doi:10.1186/s13567-020-00883-w 33597007 PMC7888150

[26] Gwinn WM, Kirwan SM, Wang SH, Ashcraft KA, Sparks NL, Doil CR, Tlusty TG, Casey LS, Hollingshead SK, Briles DE, Dondero RS, Hickey AJ, Foster WM, Staats HF. 2010. Effective induction of protective systemic immunity with nasally administered vaccines adjuvanted with IL-1. Vaccine 28:6901–6914. doi:10.1016/j.vaccine.2010.08.006 20723629 PMC2943532

[27] Meenakshi S, Kumar VU, Dhingra S, Murti K. 2022. Nasal vaccine as a booster shot: a viable solution to restrict pandemic? Clin Exp Vaccine Res 11:184–192. doi:10.7774/cevr.2022.11.2.184 35799869 PMC9200647

[28] Li L, Hao J, Jiang Y, Hao P, Gao Y, Chen J, Zhang G, Jin N, Wang M, Li C. 2023. A micro-sized vaccine based on recombinant Lactiplantibacillus plantarum fights against SARS-CoV-2 infection via intranasal immunization. Acta Pharm Sin B 13:3168–3176. doi:10.1016/j.apsb.2023.01.005 36852097 PMC9946889

[29] Sørvig E, Mathiesen G, Naterstad K, Eijsink VGH, Axelsson L. 2005. High-level, inducible gene expression in Lactobacillus sakei and Lactobacillus plantarum using versatile expression vectors. Microbiology 151:2439–2449. doi:10.1099/mic.0.28084-0 16000734

[30] Su M, Li C, Qi S, Yang D, Jiang N, Yin B, Guo D, Kong F, Yuan D, Feng L, Sun D. 2020. A molecular epidemiological investigation of PEDV in China: Characterization of co-infection and genetic diversity of S1-based genes. Transbound Emerg Dis 67:1129–1140. doi:10.1111/tbed.13439 31785090 PMC7233288

[31] Hanson G, Coller J. 2018. Codon optimality, bias and usage in translation and mRNA decay. Nat Rev Mol Cell Biol 19:20–30. doi:10.1038/nrm.2017.91 29018283 PMC6594389

[32] Eijsink VG, Brurberg MB, Middelhoven PH, Nes IF. 1996. Induction of bacteriocin production in Lactobacillus sake by a secreted peptide. J Bacteriol 178:2232–2237. doi:10.1128/jb.178.8.2232-2237.1996 8636023 PMC177930

[33] Kirchdoerfer RN, Bhandari M, Martini O, Sewall LM, Bangaru S, Yoon K-J, Ward AB. 2021. Structure and immune recognition of the porcine epidemic diarrhea virus spike protein. Structure 29:385–392. doi:10.1016/j.str.2020.12.003 33378641 PMC7962898

[34] Ouyang K, Binjawadagi B, Kittawornrat A, Olsen C, Hiremath J, Elkalifa N, Schleappi R, Wu J, Zimmerman J, Renukaradhya GJ. 2013. Development and validation of an assay to detect porcine reproductive and respiratory syndrome virus-specific neutralizing antibody titers in pig oral fluid samples. Clin Vaccine Immunol 20:1305–1313. doi:10.1128/CVI.00276-13 23784856 PMC3754528

[35] Ouyang K, Shyu D-L, Dhakal S, Hiremath J, Binjawadagi B, Lakshmanappa YS, Guo R, Ransburgh R, Bondra KM, Gauger P, Zhang J, Specht T, Gilbertie A, Minton W, Fang Y, Renukaradhya GJ. 2015. Evaluation of humoral immune status in porcine epidemic diarrhea virus (PEDV) infected sows under field conditions. Vet Res 46:140. doi:10.1186/s13567-015-0285-x 26667229 PMC4699368

[36] Tian J, Sun J, Li D, Wang N, Wang L, Zhang C, Meng X, Ji X, Suchard MA, Zhang X, Lai A, Su S, Veit M. 2022. Emerging viruses: cross-species transmission of coronaviruses, filoviruses, henipaviruses, and rotaviruses from bats. Cell Rep 39:110969. doi:10.1016/j.celrep.2022.110969 35679864 PMC9148931

[37] Li Z, Ma Z, Dong L, Yang T, Li Y, Jiao D, Han W, Zheng H, Xiao S. 2022. Molecular mechanism of porcine epidemic diarrhea virus cell tropism. mBio 13:e03739-21. doi:10.1128/mbio.03739-21 35285698 PMC9040822

[38] Niu Z, Zhang S, Xu S, Wang J, Wang S, Hu X, Zhang L, Ren L, Zhang J, Liu X, Zhou Y, Yang L, Song Z. 2023. Porcine epidemic diarrhea virus replication in human intestinal cells reveals potential susceptibility to cross-species infection. Viruses 15:956. doi:10.3390/v15040956 37112936 PMC10142432

[39] Haas J, Park E-C, Seed B. 1996. Codon usage limitation in the expression of HIV-1 envelope glycoprotein. Curr Biol 6:315–324. doi:10.1016/s0960-9822(02)00482-7 8805248

[40] Wang M, Fu T, Hao J, Li L, Tian M, Jin N, Ren L, Li C. 2020. A recombinant Lactobacillus plantarum strain expressing the spike protein of SARS-CoV-2. Int J Biol Macromol 160:736–740. doi:10.1016/j.ijbiomac.2020.05.239 32485251 PMC7260514

[41] Li L, Wang M, Hao J, Han J, Fu T, Bai J, Tian M, Jin N, Zhu G, Li C. 2021. Mucosal IgA response elicited by intranasal immunization of Lactobacillus plantarum expressing surface-displayed RBD protein of SARS-CoV-2. Int J Biol Macromol 190:409–416. doi:10.1016/j.ijbiomac.2021.08.232 34499954 PMC8421092

[42] Alias NAR, Hoo WPY, Siak PY, Othman SS, Mohammed Alitheen NB, In LLA, Abdul Rahim R, Song AA-L. 2023. Effect of secretion efficiency of mutant KRAS neoantigen by Lactococcus lactis on the immune response of a mucosal vaccine delivery vehicle targeting colorectal cancer. Int J Mol Sci 24:8928. doi:10.3390/ijms24108928 37240273 PMC10219268

[43] Zhou M, Theunissen D, Wels M, Siezen RJ. 2010. LAB-secretome: a genome-scale comparative analysis of the predicted extracellular and surface-associated proteins of lactic acid bacteria. BMC Genomics 11:651. doi:10.1186/1471-2164-11-651 21092245 PMC3017865

[44] Richard M, van den Brand JMA, Bestebroer TM, Lexmond P, de Meulder D, Fouchier RAM, Lowen AC, Herfst S. 2020. Influenza A viruses are transmitted via the air from the nasal respiratory epithelium of ferrets. Nat Commun 11:766. doi:10.1038/s41467-020-14626-0 32034144 PMC7005743

[45] Briles DE, Novak L, Hotomi M, van Ginkel FW, King J. 2005. Nasal colonization with Streptococcus pneumoniae includes subpopulations of surface and invasive pneumococci. Infect Immun 73:6945–6951. doi:10.1128/IAI.73.10.6945-6951.2005 16177374 PMC1230983

[46] Persson J, Zhang Y, Olafsdottir TA, Thörn K, Cairns TM, Wegmann F, Sattentau QJ, Eisenberg RJ, Cohen GH, Harandi AM. 2016. Nasal immunization confers high avidity neutralizing antibody response and immunity to primary and recurrent genital herpes in guinea pigs. Front Immunol 7:640. doi:10.3389/fimmu.2016.00640 28082979 PMC5183738

[47] Mabbott NA, Donaldson DS, Ohno H, Williams IR, Mahajan A. 2013. Microfold (M) cells: important immunosurveillance posts in the intestinal epithelium. Mucosal Immunol 6:666–677. doi:10.1038/mi.2013.30 23695511 PMC3686595

[48] Surve MV, Lin B, Reedy JL, Crossen AJ, Xu A, Klein BS, Vyas JM, Rajagopal J. 2023. Single-cell transcriptomes, lineage, and differentiation of functional airway microfold cells. Am J Respir Cell Mol Biol 69:698–701. doi:10.1165/rcmb.2023-0292LE 38038398 PMC10704116

[49] Lavelle EC, Ward RW. 2022. Mucosal vaccines - fortifying the frontiers. Nat Rev Immunol 22:236–250. doi:10.1038/s41577-021-00583-2 34312520 PMC8312369

[50] O’Neal CM, Clements JD, Estes MK, Conner ME. 1998. Rotavirus 2/6 viruslike particles administered intranasally with cholera toxin, Escherichia coli heat-labile toxin (LT), and LT-R192G induce protection from rotavirus challenge. J Virol 72:3390–3393. doi:10.1128/JVI.72.4.3390-3393.1998 9525668 PMC109829

[51] Van Oirschot JT, De Leeuw PW. 1985. Intranasal vaccination of pigs against Aujeszky’s disease. 4. Comparison with one or two doses of an inactivated vaccine in pigs with moderate maternal antibody titres. Vet Microbiol 10:401–408. doi:10.1016/0378-1135(85)90022-7B2 2996212

[52] Zhang L, Tian X, Zhou F. 2007. Intranasal administration of CpG oligonucleotides induces mucosal and systemic Type 1 immune responses and adjuvant activity to porcine reproductive and respiratory syndrome killed virus vaccine in piglets in vivo. Int Immunopharmacol 7:1732–1740. doi:10.1016/j.intimp.2007.09.012 17996683

[53] Du B, Fu Y, Han Y, Sun Q, Xu J, Yang Y, Rong R. 2023. The lung-gut crosstalk in respiratory and inflammatory bowel disease. Front Cell Infect Microbiol 13:1218565. doi:10.3389/fcimb.2023.1218565 37680747 PMC10482113

[54] Deepika G, Charalampopoulos D. 2010. Surface and adhesion properties of lactobacilli. Adv Appl Microbiol 70:127–152. doi:10.1016/S0065-2164(10)70004-6 20359456

[55] Martin JT, Hartwell BL, Kumarapperuma SC, Melo MB, Carnathan DG, Cossette BJ, Adams J, Gong S, Zhang W, Tokatlian T, Menis S, Schiffner T, Franklin CG, Goins B, Fox PT, Silvestri G, Schief WR, Ruprecht RM, Irvine DJ. 2021. Combined PET and whole-tissue imaging of lymphatic-targeting vaccines in non-human primates. Biomaterials 275:120868. doi:10.1016/j.biomaterials.2021.120868 34091299 PMC8325633

[56] Yogev N, Frommer F, Lukas D, Kautz-Neu K, Karram K, Ielo D, von Stebut E, Probst H-C, van den Broek M, Riethmacher D, Birnberg T, Blank T, Reizis B, Korn T, Wiendl H, Jung S, Prinz M, Kurschus FC, Waisman A. 2012. Dendritic cells ameliorate autoimmunity in the CNS by controlling the homeostasis of PD-1 receptor+ regulatory T cells. Immunity 37:264–275. doi:10.1016/j.immuni.2012.05.025 22902234

[57] Mohamadzadeh M, Duong T, Sandwick SJ, Hoover T, Klaenhammer TR. 2009. Dendritic cell targeting of Bacillus anthracis protective antigen expressed by Lactobacillus acidophilus protects mice from lethal challenge. Proc Natl Acad Sci USA 106:4331–4336. doi:10.1073/pnas.0900029106 19246373 PMC2647975

