# Supplementary material for: A novel cross-species vaccine design and nasal immunization strategy based on recombinant Lactiplantibacillus plantarum expressing PEDV tS1
Source: Appl Environ Microbiol. 2026 May 11;92(6):e00386-26. doi: 10.1128/aem.00386-26 (PMC13274380; doi:10.1128/aem.00386-26)
Supplement: Fig. S1 — Virus neutralizing activity of serum, feces, and NLF. [file aem.00386-26-s0001.docx]

Supplementary Materials


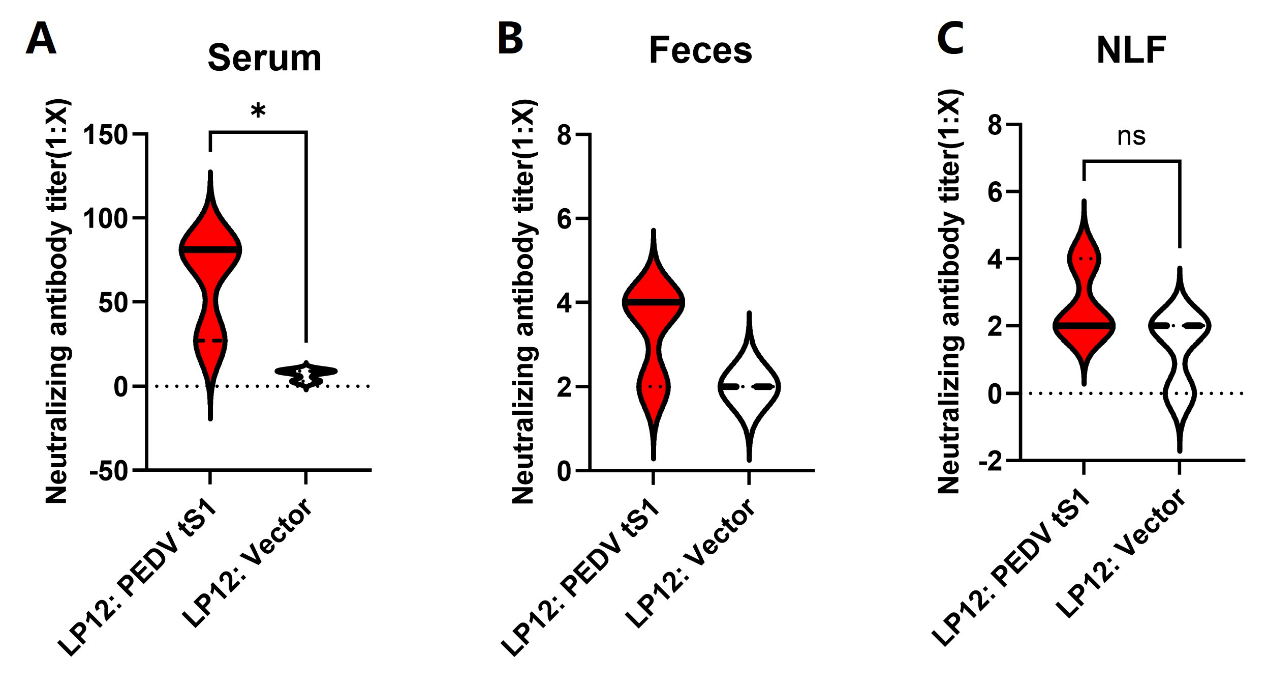


Supplementary Figure 1

Virus neutralizing activity of serum (A), feces(B), and NLF (C)
